# Comparison of the Degradability of Hyaluronic Acid by Ovine and Recombinant Human Hyaluronidase

**DOI:** 10.1093/asj/sjaf127

**Published:** 2025-06-30

**Authors:** Tyler Safran, Roy Khalaf, Andrei Metelitsa, Julie Woodward, Andreas Nikolis

## Abstract

**Background:**

The mainstay of treatment for adverse events to hyaluronic acid filler is the use of hyaluronidase (HYAL); however, the dose and dilution are not standardized.

**Objectives:**

The objective of this study was to examine differential dilutions and concentrations of HYAL, and to compare the effectiveness of ovine and human HYAL.

**Methods:**

Fillers were selected for study based on a variety of rheologic factors. A 0.2-mL dose of product was selected for use based on previous studies. Degradation was assessed by comparing both ovine and recombinant HYAL over a range of concentrations and dilutions.

**Results:**

In the 2:1 dilution group, Restylane Lidocaine (Galderma, Lausanne, Switzerland) was degraded by 100 U of ovine HYAL after 50 minutes. No other filler was completely degraded; Restylane Lyft (Galderma) was partially degraded after 1 hour of treatment with 100 U of HYAL. In both the 3:1 and 4:1 dilution groups, Restylane Shaype (Galderma) and Restylane Lyft fillers were most susceptible to degradation, dissolving within 30 minutes with 100 U of recombinant HYAL and within 40 minutes with 100 U of ovine HYAL. Juvéderm and RHA4 fillers were the most resistant, requiring 300 U of HYAL for degradation within 1 hour. Beyond a 3:1 dilution ratio, no further improvement was observed.

**Conclusions:**

This study demonstrates that 300 U of hyaluronidase is sufficient to degrade 0.2 mL of the most resistant hyaluronic acid fillers within 1 hour. Importantly, a minimum dilution of 3:1 should be used to provide adequate fluid for dissolving filler. Ovine HYAL appears to be just as effective as recombinant HYAL in terms of dissolving product.

The use of hyaluronic acid (HA)-based fillers for facial shaping and rejuvenation has continued to grow year over year.^1^ As their use increases, so does innovation in product type, rheology, and injection technique. Given the widespread use of HA by many varieties of practitioners, it is of paramount importance to understand management of adverse events. Although such events are rare, HA fillers are associated with both vascular and nonvascular adverse events of varying degrees of severity that require treatment.^2^ The mainstay of treatment is the use of hyaluronidase (HYAL), an endoglycosidase that breaks down HA into monosaccharides by cleaving its glycosidic bonds.^3^ In North America, 3 HYAL formulations are currently available: Vitrase (ovine testicular HYAL; Bausch & Lomb, Laval, Canada), Amphadase (bovine HYAL; Amphastar Pharmaceuticals, Rancho Cucamonga, CA), and Hylenex (recombinant human HYAL; Halozyme Therapeutics, San Diego, CA).^4^

For HYAL to dissolve a HA filler, it must interact with its binding sites within the HA itself.^3^ The reaction of a filler to HYAL depends on many factors, including but not limited to the HA concentration, the number of crosslinks, and the form of the filler.^3^ HYAL is rapidly degraded and deactivated in the body, with some reports demonstrating a loss of in vivo effect after 3 to 6 hours.^5^ Therefore, to dissolve a HA filler, enough HYAL must be injected close to the filler. Both animal-derived and recombinant human HYAL have been shown to efficiently degrade HA.^6,7^ Differences in their enzymatic structure and their immunogenicity may impact their efficacy and safety profiles.^8^ While animal HYAL has demonstrated efficacy, bovine-derived HYAL has been associated with hypersensitivity reactions and, in rare cases, anaphylaxis.^8,9^

Currently there is a lack of standardization and knowledge of HYAL dosing, dilutions, administration, and treatment algorithms. To answer this question, many authors have performed both in vitro and in vivo studies examining the various HA products available and their response to varying concentrations of HYAL (both animal and human derived).^7,10^ These studies mainly examined how linearly increasing units of HYAL dissolved fixed amounts of HA. Unsurprisingly, increasing the HYAL dose yielded more robust degradation of product, but this increase in HYAL was also accompanied with an increase in the overall volume of the reconstituted HYAL.^7,10^ Additionally, these studies all either manually agitated the filler/HYAL mixture by stirring or by direct injection. In vivo, this becomes more of a challenge without advanced ultrasound capabilities or exact knowledge of product location.

No study to date, however, has examined how HA fillers react and degrade when treated with varying ratios of HYAL/saline to filler (ie, liquid-to-solid ratios). Additionally, no study has compared the efficacy of ovine to human recombinant HYAL in this context. The goal of this study was to examine how changing the ratio of HYAL/saline to filler by varying the HYAL concentration influences the degradation of a range of HA fillers, and to compare the efficacy of ovine and human recombinant HYAL at different dilutions.

## METHODS

Multiple experiments were designed to test the efficacy of ovine HYAL, to compare ovine and human HYAL, and to determine optimal dilutions of HYAL to achieve dissolution of HA. These studies were performed between September and November 2024.

### Experiment 1 (1:1 and 2:1 dilutions)

The first experimental study examined the response of 5 HA gels to lower dilutions of ovine HYAL. Five fillers—Restylane Shaype (R_S_; NASHA-HD, Galderma, Lausanne, Switzerland), Restylane Lyft (R_L_; NASHA, Galderma), Restylane Lidocaine (R_Lido_; NASHA, Galderma), Revanesse Sculpt+ (R_SC_; Prollenium, Richmond Hill, Canada) and Juvederm Volux (J_VX_; VYCROS, Abbvie, North Chicago, IL)—were selected for study to provide a variety of rheologies. These gels were selected based on previous studies, selecting products that were easily degraded vs those that were not.^11^ Table 1 includes all product information.

**Table 1.** Characteristics and Indications of Various Hyaluronic Acid Fillers

HA filler	Manufacturing technology	Concentration of hyaluronic acid (mg/mL)	*G*'^12^	*G*''^12^	Tan delta^12^	Cohesivity/Normal force^12^	Maximum water uptake^12^	Indications for use
R_L_	NASHA	20	977	198	0.203	32	<100	Moderate-severe facial folds and wrinkles^13^
R_Lido_	NASHA	20	544	NA	NA	NA	NA	
R_S_	NASHA	20	NA	NA	NA	NA	NA	Skin smoothness and appearance^13^
R_SC_	NA	NA	NA	NA	NA	NA	NA	
R_H_	RHA	23	346	62	0.179	115	366	Injection in deep dermis to superficial subcutaneous tissue
B_V_	CPM	26	438	103	0.235	97	370	Restoration of facial volume^13^
J_VO_	Vycross	20	398	41	0.103	40	227	Restores volume of the face Deep dermis, subcutaneously^14^
J_VX_	Vycross	25	665	49	0.074	93	253	Injection in deep dermis Jawline and chin contouring

B_V_, Belotero Volume; CPM, cohesive polydensified matrix; HA, hyaluronic acid; J_VO_, Juvederm Voluma; J_VX_, Juvederm Volux; NA, not applicable; NASHA, non-animal stabilized hyaluronic acid; R_H_, RHA, resilient hyaluronic acid; RHA4; R_L_, Restylane Lyft; R_Lido_, Restylane Lidocaine; R_S_, Restylane Shaype; R_SC_, Revanesse Sculpt+.

The dosage of HA was chosen based on previous in vitro studies to provide a comparison.^11^ As a result, 0.2 mL of filler was selected. Degradation was measured every 10 minutes until complete degradation to a limit of 60 minutes. This process was repeated with increased dosages of 20, 60 and 100 U of HYAL (3 groups). With a fixed HA volume of 0.2 mL, the volumes of HYAL and normal saline (NS) required to achieve effective degradation were determined while maintaining the ratio of HYAL/NS to filler in the group. To do so, liquid-to-solid ratios of 1:1 and 2:1 were selected for this group. Normal saline alone was used as a control group, respecting the same dilutions (fourth group). Ovine HYAL (1500 U/mL) was utilized to be able to accurately dilute the solution to the appropriate ratios.

All products were injected with low extrusion force into Petri dishes via a 25G needle by the same individual. HYAL solutions were created and placed in perifluid without injecting it intrasubstance and without stirring.

### Experiment 2

The second experimental study examined the response of 7 HA gels to 2 kinds of HYAL available in North America, recombinant human HYAL and ovine HYAL, at higher dilutions. Based on previous findings in the literature,^11^ two non-animal stabilized hyaluronic acid fillers, R_S_ and R_L_, were selected, as well as the 5 most resistant fillers identified: R_SC_, Belotero Volume (B_V_; Merz Aesthetics, Raleigh, NC), RHA4 (R_H_; Teoxane, Geneva, Switzerland), Juvederm Voluma (J_VO_, Abbvie) and J_VX_. Table 1 includes all filler information.

Two experiments were designed to assess degradation of each respective HA filler by each substrate of HYAL, as well as different doses and dilutions. In this study, NS was used as a control, as well as HYAL Hylenex 150 U/mL and ovine HYAL 150 U/mL. The same injection protocol as Experiment 1 was utilized.

Like the first experiment, each group was designed to maintain precise liquid-to-solid ratios of 3:1 and 4:1. Four groups were utilized to ensure consistency in volume control.

Again, both experiments were completed using both human and ovine HYAL. Table 2 lists all dilutions and filler quantities.

**Table 2.** Dilutions of HYAL and Study Group Make-up

Group number	1:1 Volume ovine HYAL (1500 U/mL)	2:1 Volume ovine HYAL (1500 U/mL)	3:1 Volume human and ovine (150 U/mL)	4:1 Volume human and ovine (150 U/mL)
1 (Saline)	0.2 mL HA + 0.2 mL NS	0.2 mL HA + 0.4 mL NS	0.2 mL HA + 0.66 mL NS	0.2 mL HA + 0.83 mL NS
2 (20 U HYAL)	0.2 mL HA + 0.013 mL HYAL + 0.187 mL NS	0.2 mL HA + 0.013 mL HYAL + 0.374 mL NS	0.2 mL HA + 0.13 mL HYAL + 0.53 mL NS	0.2 mL HA + 0.13 mL HYAL + 0.70 mL NS
3 (60 U HYAL)	0.2 mL HA +0.04 mL HYAL + 0.16 mL NS	0.2 mL HA +0.04 mL HYAL + 0.32 mL NS	0.2 mL HA +0.39 mL HYAL + 0.27 mL NS	0.2 mL HA +0.39 mL HYAL + 0.44 mL NS
4 (100 U HYAL)	0.2 mL HA + 0.0667 mL HYAL + 0.1333 mL NS	0.2 mL HA + 0.0667 mL HYAL + 0.267 mL NS	0.2 mL HA + 0.67 mL HYAL	0.2 mL HA + 0.67 mL HYAL + 0.17 mL NS

HA, hyaluronic acid; HYAL, hyaluronidase; NS, normal saline.

### Experiment 3: Higher Dose Experiment

In the event of nondegradability of a filler, a subsequent trial was conducted with higher doses of HYAL. To maintain the same ratios (3:1 and 4:1), only ovine HYAL (1500 U/mL) was used. The dose of HYAL was incrementally increased by 50 units/per experiment. The same injection and HYAL placement procedures as used in the other experiments were followed.

### Macroscopic Photography

Photography of the HA fillers were taken immediately postinjection (1, 3, and 5 minutes), and at intervals of 10 minutes for 1 hour, using a camera with a macroscopic lens. Each photograph was taken with the same lighting and at the same distance from the sample.

### Microscopic Assessment

In addition to the macroscopic photography, high-powered microscopy was utilized by the evaluators to determine if any residual filler was present (see next section).

### Degradation Assessment

To evaluate the degradation of HA fillers, a visual assessment was conducted using both macroscopic photography and high-powered microscopy at predetermined time intervals (30 minutes, 40 minutes, 50 minutes, and 1 hour) after the injection of HYAL by 3 independent reviewers (2 board-certified plastic surgeons and 1 oculoplastic surgeon). The primary outcome measured was the visible dissolution (complete degradation) or reduction (partial degradation) in the volume of the HA filler at each time point. Complete degradation was defined as no evidence of physical filler remaining. No stirring was performed, as seen in previous studies, because it is physically impossible to manipulate filler in vivo. A Fleiss's kappa analysis was performed to assess the level of agreement among the 3 evaluators in determining whether the product was degraded at each time point. This statistical test was selected given the binary (yes/no) input of each reviewer at the selected time intervals.

## RESULTS

### Low Dilutions (1:1 and 2:1)

**Figure 1.**
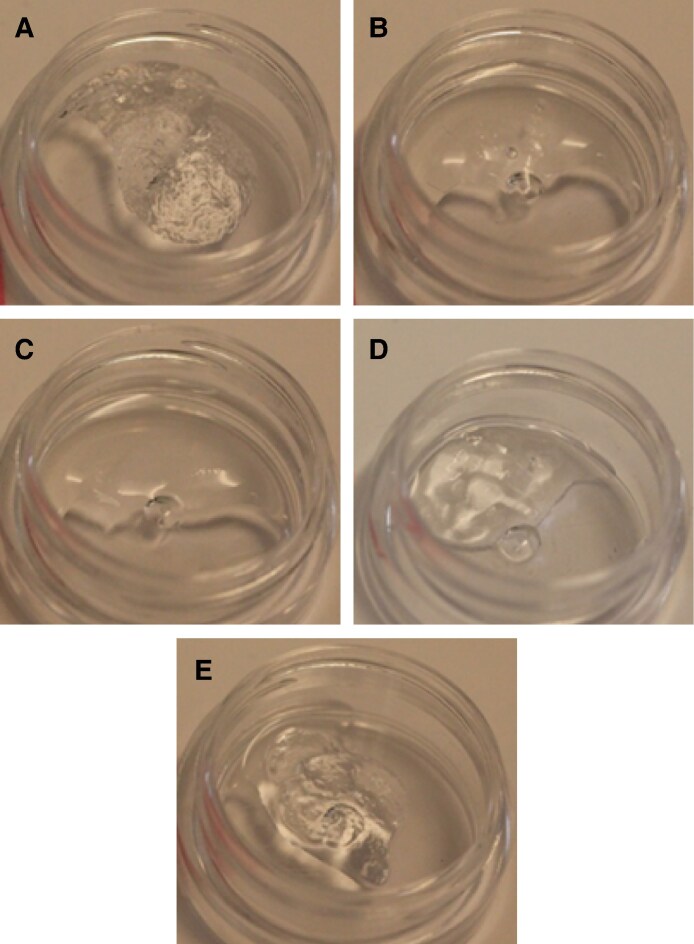
Comparison of the degradability of 0.2 mL of 5 different hyaluronic acid fillers by 100 U of ovine hyaluronidase at a liquid-to-solid ratio of 1:1: (A) Restylane Shaype, (B) Restylane Lyft, (C) Restylane Lidocaine, (D) Revanesse Sculpt+, and (E) Juvederm Volux.

For the 1:1 ratio trial, at time points of 30 minutes, 40 minutes, 50 minutes, and 1 hour, no degradation (complete or partial) was seen in any of the 5 fillers with up to 100 U of ovine HYAL. Additionally, no degradation was seen in any of the control samples treated with pure NS (Figure 1).

**Figure 2.**
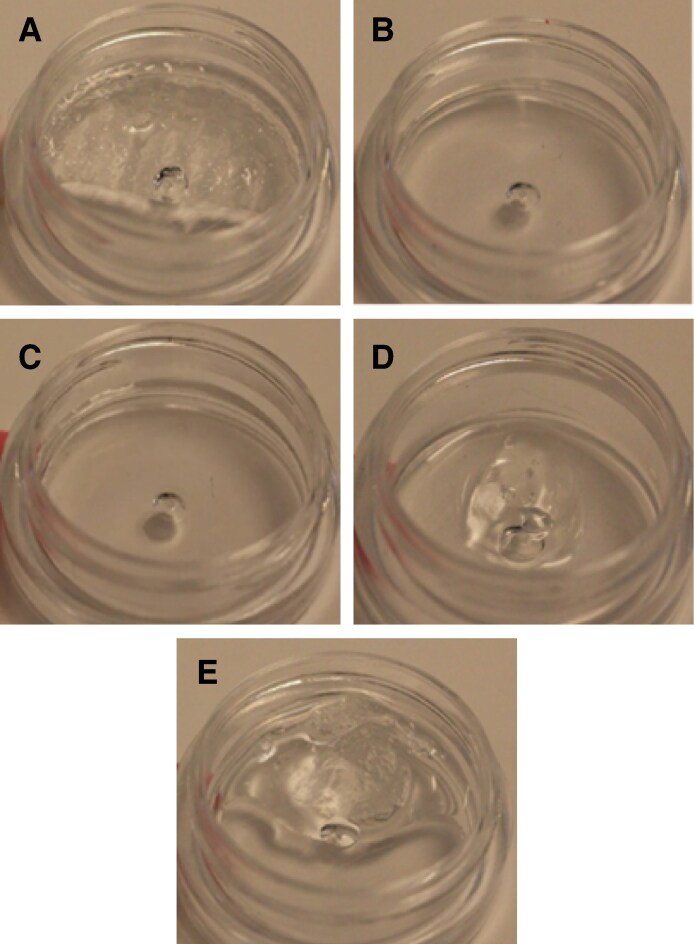
Comparison of the degradability of 0.2 mL of 5 different hyaluronic acid fillers by 100 U of ovine hyaluronidase at a liquid-to-solid ratio of 2:1: (A) Restylane Shaype, (B) Restylane Lyft, (C) Restylane Lidocaine, (D) Revanesse Sculpt+, and (E) Juvederm Volux.

For the 2:1 ratio, no degradation was seen at 30 and 40 minutes in any dilution. By 50 minutes, R_L_ was totally degraded with 100 U of ovine HYAL. No other complete degradation of fillers was noted. In terms of partial degradation, R_L_ was noted to undergo partial degradation by 1 hour with 100 U of HYAL (Figure 2).

### Higher Dilutions (3:1 and 4:1) and Comparison of Substrates

**Figure 3.**
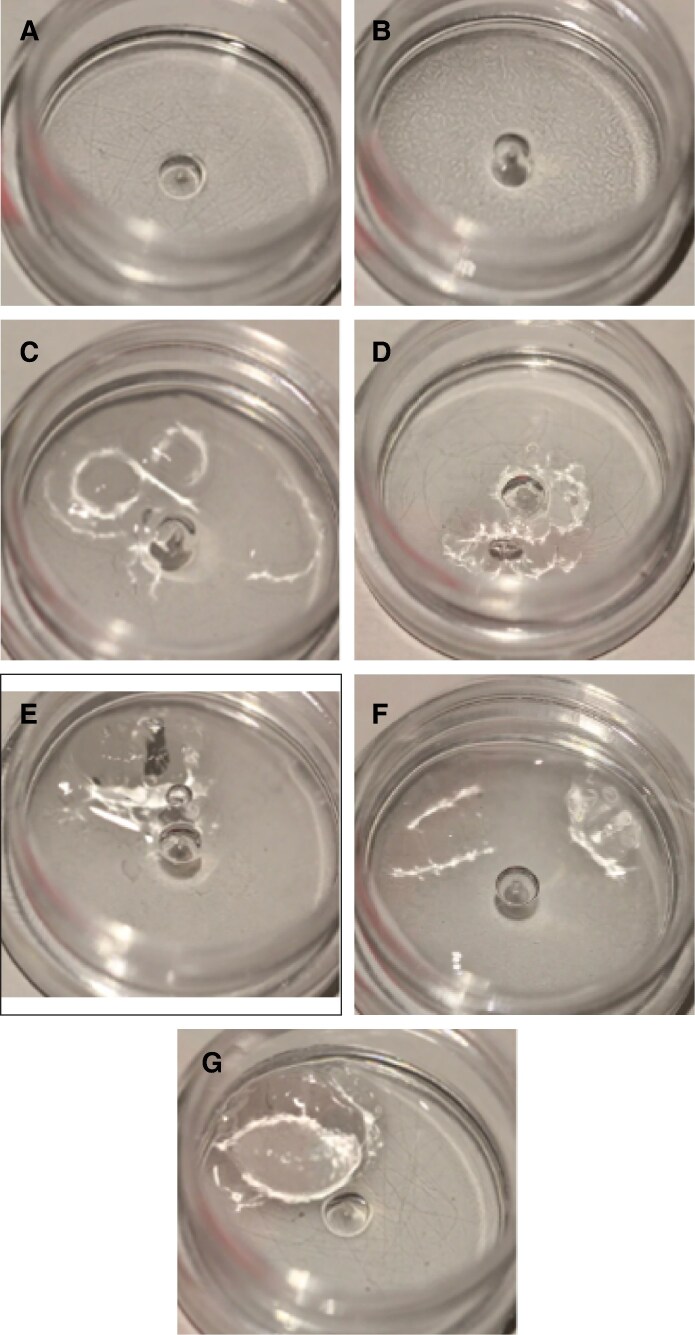
Comparison of the degradability of 0.2 mL of 7 hyaluronic acid fillers by 100 U of ovine hyaluronidase at 40 minutes: (A) Restylane Shaype (R_S_), (B) Restylane Lyft (R_L_), (C) Revanesse Sculpt+ (R_SC_), (D) RHA4 (R_H_), (E) Belotero Volume (B_V_), (F) Juvederm Voluma (J_VO_), and (G) Juvederm Volux (J_VX_).

In both the 3:1 and 4:1 experiments using ovine HYAL, R_L_ and R_S_ were the first to dissolve, achieving complete degradation at 40 minutes following the injection of 100 U of ovine HYAL. No degradation was observed for the remaining 5 HA fillers after 1 hour of observation with 100 U. Figure 3 illustrates the dissolution rates of all 7 HA fillers at 40 minutes following injection of ovine HYAL.

**Figure 4.**
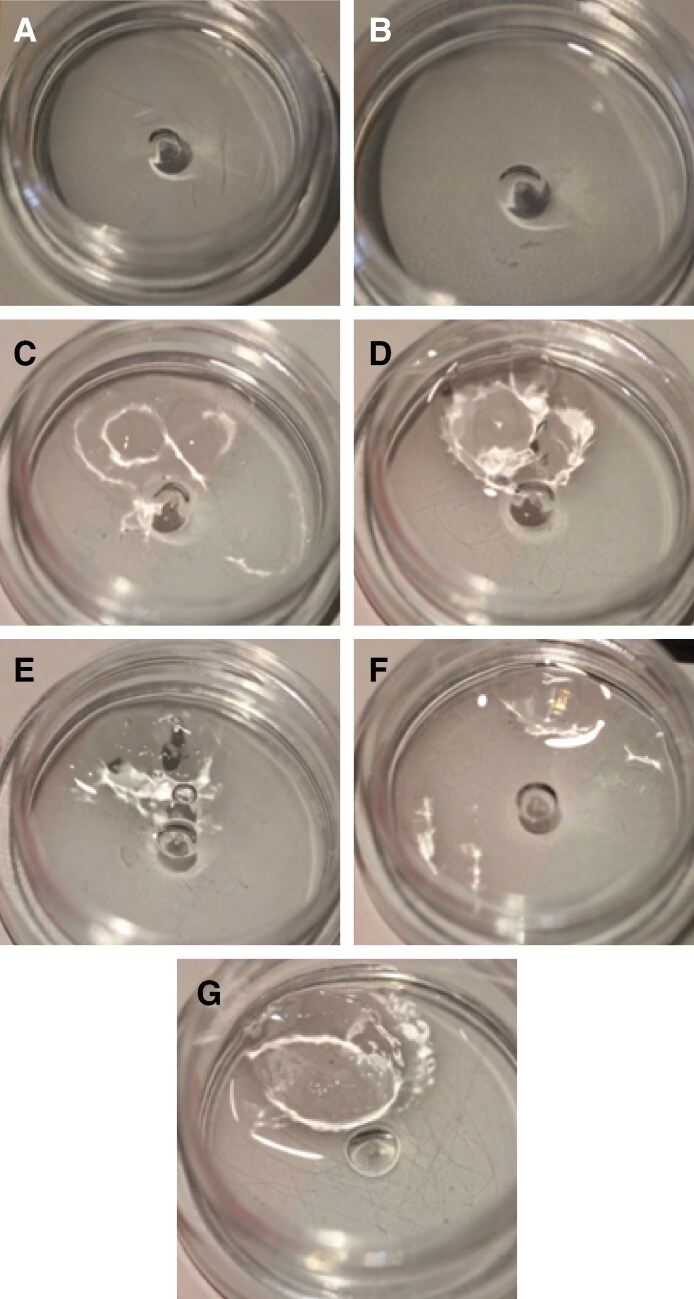
Comparison of the degradability of 0.2 mL of 7 hyaluronic acid by 100 units of recombinant human hyaluronidase at 60 minutes: (A) Restylane Shaype (R_S_), (B) Restylane Lyft (R_L_), (C) Revanesse Sculpt+ (R_SC_), (D) RHA4 (R_H_), (E) Belotero Volume (B_V_), (F) Juvederm Voluma (J_VO_), and (G) Juvederm Volux (J_VX_).

For recombinant HYAL, in both the 3:1 and 4:1 groups, R_L_ was degraded first by 60 U at 30 minutes, while R_S_ required 100 U of recombinant HYAL and was degraded after 1 hour. The remaining 5 fillers did not show signs of degradation after 1 hour of observation with up to 100 U. Figure 4 illustrates the dissolution rates of all 7 HA fillers with human HYAL.

### Higher-Concentration Experiment

For this phase, the 5 fillers that had not been degraded by 100 U of either ovine or recombinant HYAL were injected with 250 and 300 U of ovine HYAL. This resulted in Group 5 (250 U: 0.167 mL HYAL + 0.493 mL NS) and Group 6 (300 U: 0.2 mL HYAL + 0.46 mL NS) in Experiment 1 (3:1), and Group 5 (250 U: 0.167 mL HYAL + 0.663 mL NS) and Group 6 (300 U: 0.2 mL HYAL + 0.63 mL NS) in Experiment 2 (4:1).

**Figure 5.**
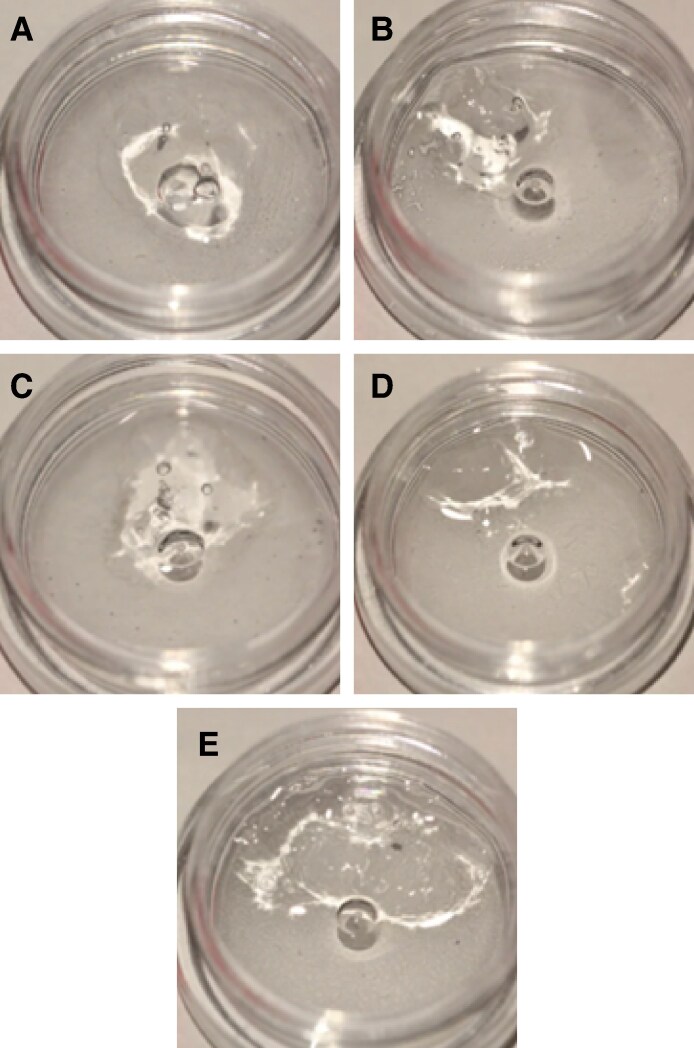
Comparison of the degradability of 0.2 mL of 5 hyaluronic acid fillers by 250 U of recombinant human hyaluronidase at 60 minutes: (A) Revanesse Sculpt+ (R_SC_), (B) RHA4 (R_H_), (C) Belotero Volume (B_V_), (D) Juvederm Voluma (J_VO_), and (E) Juvederm Volux (J_VX_).

**Figure 6.**
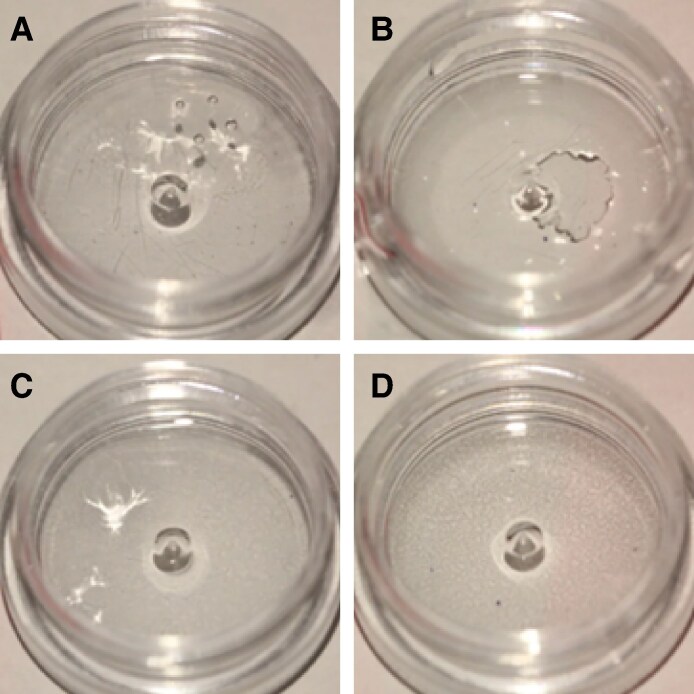
Comparison of degradability of the 0.2 mL of four hyaluronic acid fillers by 300 U of recombinant human hyaluronidase at 60 minutes: (A) RHA4 (R_H_), (B) Belotero Volume (B_V_), (C) Juvederm Voluma (J_VO_), and (D) Juvederm Volux (J_VX_).

The results are summarized in Table 3. Following the injection of 250 and 300 U of ovine HYAL in both the 3:1 and 4:1 groups, R_SC_ and B_V_ were the most susceptible to degradation, with both dissolving by 40 minutes. J_VO_ was degraded by 50 minutes by 300 U of HYAL, while J_VX_ was degraded after 1 hour with 300 U of HYAL. Lastly, R_H_ was also degraded after 1 hour by 300 U of HYAL. All results were seen in both the 3:1 and 4:1 groups (Figures 5, 6).

**Table 3.** Time to Degradation of Various HA Fillers by Different Concentrations of Ovine and Recombinant HYAL

HA filler	Ovine HYAL (100 U)	Recombinant HYAL (100 U)	Ovine HYAL (250 U)	Ovine HYAL (300 U)	Fleiss’s kappa
R_L_	40 minutes	30 minutes (60 U)	Not tested	Not tested	1
R_S_	40 minutes	1 hour	Not tested	Not tested	1
R_SC_	No degradation	No degradation	40 minutes	40 minutes	1
R_H_	No degradation	No degradation	No degradation	1 hour	1
B_V_	No degradation	No degradation	No degradation	40 minutes	1
J_VO_	No degradation	No degradation	No degradation	50 minutes	1
J_VX_	No degradation	No degradation	No degradation	1 hour	1

All values represent the time to visible degradation as assessed independently by 3 raters. Fleiss's kappa = 1.0, indicating complete interrater agreement. B_V_, Belotero Volume; HA, hyaluronic acid; HYAL, hyaluronidase; J_VO_, Juvederm Voluma; J_VX_, Juvederm Volux; R_H_, RHA4; R_L_, Restylane Lyft; R_S_, Restylane Shaype; R_SC_, Revanesse Sculpt+.

### Fleiss Kappa Score

All 3 reviewers provided identical ratings at all time points, indicating complete agreement across all assessments. As a result, the Fleiss kappa score was calculated to be 1, reflecting perfect interrater agreement.

## DISCUSSION

This study was successful at demonstrating a difference in degradation of HA products based on differences in dilution and dose of the HYAL (liquid-to solid ratio), with no further subsequent improvement seen after a 3:1 dilution. Interestingly, all reviewers provided identical ratings (complete concordance) for every time point, indicating complete agreement across all assessments. No filler was dissolved in the 1:1 group even at concentrations of 100 U of ovine HYAL. This differed from the 2:1 group with 100 U of HYAL successfully dissolving R_Lido_ and partially degrading R_L_. Interestingly, previous studies have demonstrated degradation of both R_L_ and R_Lido_ products with less than 100 U of product.^11^ Park et al, however, used volumes to a maximum of 0.9 mL (120 U of human recombinant HYAL) which represents a liquid-to-solid ratio of 4.5:1.^11^ Interestingly, in another study, when lower ratios (0.1 mL with 0.15 mL of HYAL reconstituted) were considered, only partial degradation of the products was visualized, and more degradation of product was visualized with an increased concentration 40 U of HYAL, but that also included a larger liquid-to-filler ratio of 3:1.^15^ Lastly, in terms of manipulation of product, one study also used smaller volumes of reconstituted HYAL (100 U in 0.05 mL) to degrade 0.1 mL of product (ratio, 0.5), however only partial degradation of R_L_ was seen after stirring of the HYAL/filler mixture (*P* < .001).^16^

The study was also successful at demonstrating similar effectiveness when using either human recombinant or ovine HYAL. Both were able to dissolve the same products in similar timeframes. In terms of individual products, R_L_ and R_S_ were the most susceptible to degradation by both recombinant and ovine HYAL. Specifically, 100 U of ovine HYAL degraded R_S_ within 30 minutes, whereas 100 U of recombinant HYAL took 1 hour to achieve the same result. One study demonstrated that ovine HYAL significantly dissolved R_Lido_ more effectively within 4 days postinjection, compared with recombinant human HYAL.^12^ In contrast, Rao et al observed no difference in the degradation of Restylane, Juvéderm, or Belotero by either recombinant or ovine HYAL, despite using similar concentrations of Restylane.^13^

The fillers that were the most resistant to degradation in this study were R_H_, J_VX_, and J_VO_. This observation aligns with the findings of Park et al, who ranked J_VX_ as the most resistant.^11^ Despite the common belief that a higher *G*' correlates with greater resistance to degradation, the fillers that had higher *G*' values, such as R_L_ and R_S_, were the easiest to degrade in the present study. In this study, both R_H_ and J_VX_ demonstrated similar levels of resistance, being degraded at 300 U of ovine HYAL 1 hour postinjection. Although their *G*' values were lower than that of R_L_, other factors likely contributed to their resistance. This can be partially attributed to their higher concentrations of HA, 26 mg/mL for J_VX_ and 23 mg/mL for R_H._ In this study, B_V_ was more easily degraded by ovine HYAL than J_VX_ and R_H_. This differs from a study by Buhren et al who reported that B_V_ maintained its form better than Juvéderm and Restylane fillers after degradation by ovine HYAL, suggesting that B_V_ may exhibit greater resistance.^14^

Overall, this study's findings suggest that 300 U of HYAL is sufficient to degrade 0.2 mL of the most resistant HA fillers within 1 hour. It is recommended that clinicians consider this dose to achieve efficient degradation of HA fillers in under 1 hour. Of note, the 300 U of HYAL was administered in a 3:1 ratio. If a standard 150 U/mL reconstitution is used, for 0.2 mL of filler, this would surpass the 3:1 ratio, and thus provide ample dilution.

Previous consensus and review articles have suggested that to treat vascular events, injecting the area with around 400 to 600 IU of HYAL is required to adequately bathe the HA and provide ample dissolution to restore blood flow.^17^ One consensus panel on the management of adverse events in HA injections found that 93% of participants view the Delorenzi protocol to be the gold standard for vascular events.^17^ The Delorenzi protocol involves HYAL dosing based on the volume of ischemic tissue, with hourly repeated dosing to maintain high concentrations of HYAL throughout the ischemic zone.^18^ This new theory was based on the idea that an affected vessel must be sufficiently bathed with the enzyme solution to be able to work. An example given is for a low-volume vascular embolic event (defined as <0.1 mL): a usual dose would be 3 mL of HYAL (about 450 U in a reconstituted dose) and this dose increases with the size of the affected area.^18^ This represents a 30:1 ratio of liquid (solution) to solid (filler), much greater than seen in the present study.

With the novel use of ultrasound-based devices, delivery of HYAL to the affected area may be more reliable, especially for nonvascular correction, but nevertheless no clear consensus exists on dosage or dilution. Additionally, as demonstrated in previous studies, intrafiller injection of HYAL may increase the ability to dissolve filler using less HYAL when compared with perifiller injection.^11^ Unfortunately, ultrasound technology is not carried in a vast majority of clinics where HA filler is administered and its use involves a learning curve.

Understanding appropriate dosing and dilution is critical, especially in nonvascular cases, given the possible side effects from large doses of HYAL. One adverse event that has been described is referred to as post-HYAL syndrome, which may include hollowing of the facial tissues, loss of skin elasticity, or discoloration of the skin.^19^ Post-HYAL syndrome was described in a 90-patient case series treated with HYAL over a 4-year period for swelling (52%), lumpiness (20%), and filler dissolution prior to blepharoplasty (17%).^19^ The study did conclude, however, that post-HYAL syndrome may be related to previous filler volume and duration, rather than the dose of HYAL used. Variable doses were used: 150 IU/mL in 66% patients, 75 IU/mL in 31%, 37.5 IU/mL in 3%, and 100 IU/mL in 1%. Additionally, HYAL can be associated with an allergic reaction, although this is rare.^20^ Previous studies have described allergic reactions, hypersensitivity, and angioedema related to the use of ovine HYAL. The incidence of allergic reactions has been reported to be 0.05% to 0.69%, and urticaria and angioedema have also been reported to occur at an even lower frequency.^3^ One review found that these allergic reactions can present up to 120 hours after injection.^21^ If warranted, a skin test can be performed with 3 IU of HYAL.^3^ Consensus panels have not demonstrated much utility in previous skin testing with the incidence of allergic reaction being so low.^17,20^ Of note, injection of ovine HYAL should be avoided in patients who are allergic to bovine collagen and bee stings due to possible cross-reactivity.

It is important to note that HYAL has previously been added in varying quantities to local anesthesia to help with tissue dispersion.^22^ It is therefore hypothesized that, in vivo, even lower ratios may be successful at partially degrading product if the HYAL can be adequately spread around the filler, although this has not been demonstrated. Additionally, given the pain associated with filler dissolving, some authors have advocated for the use of lidocaine to be added to the HYAL mixture.^23^ Although some authors argue this may change the pH of the surrounding tissue, rendering HYAL less effective, similar to what is seen in the context of infection and biofilm, it may contrarily also render the mixture more dilute and increase the ratio of solid to liquid.^24^

In terms of aesthetic correction or for partial degradation, previous studies have demonstrated that lower concentrations of HYAL (20 U) may be as effective as higher concentrations (40 U), with a general rule of 20 U for 4 to 6 mg of HA.^25^ Regardless, when correcting for nodules or the Tyndall effect, less can be administered and the patient may return for further treatment. The same, unfortunately, cannot be said about vascular events. One panel survey, which surveyed 264 healthcare professionals, found that the majority used a concentration of 500 to 750 IU/mL for vascular events and 150 to 250 IU/mL for nonvascular adverse events, but there was wide variability in the group.^26^ Additionally, 5 percent of respondents admitted to observing events related to the HYAL itself, including swelling, anaphylaxis, skin laxity, and soft tissue atrophy.^26^

This study demonstrated that the amount of fluid injected (ratio of HYAL mixture to filler) may play a larger role than previously expected, especially in patients who are referred for aesthetic correction. This trial demonstrates that the liquid-to-solid ratio is likely as important as the concentration of HYAL, especially when injected into the surrounding tissues. This effect is further compounded when vascular injection is seen, as the HYAL needs to not only penetrate the filler, but also the vessel wall. One ex vivo study examined portions of the anterior jugular vein and facial artery from neck dissections. The vein and artery specimens were filled with 25 mg/mL of crosslinked HA filler. Each specimen was soaked in 0.5 mL of HYAL (300 IU/mL), in its own test tube, for 4 hours, after which the remaining HA volume was found to be 0.02 mL in the vein segment and 0.002 mL in the artery segment.^27^ While the efficacy of intravascular injection of HYAL is still up for debate, with some injectors feeling that this may in fact propagate the embolic filler forward, it is still of upmost importance to ensure an adequate amount of HYAL is injected perivascularly to help with clot breakdown and restoration of flow.

Clinically, these findings provide a foundation for future trials to explore various concentrations, ratios, and filler types to better understand the impact of ratio on filler dissolution. This is particularly relevant when examining different formulations of HYAL. For example, Hylenex recombinant is supplied as a 150 U/mL vial, which may require multiple vials for certain fillers. In contrast, ovine-derived formulations are often available in 1500 U/vials, allowing for easier storage and dilution. In aesthetic adverse events, this could result in more effective dissolving with fewer side effects. In ischemic events, it may facilitate better resolution of embolism and faster restoration of blood flow.

### Limitations

This study is not without limitations. First, considering the reliance on visual assessments for degradation, there was potential for subjectivity, despite attempts at using multiple reviewers. Second, only ovine HYAL was used for higher-concentration experiments due to limited availability of higher concentrations of recombinant HYAL; this may introduce variability that limits direct comparison at these elevated HYAL doses. Moreover, the controlled experimental study may not fully replicate real-world conditions. Factors such as individual patient variability, tissue type, and injection technique could affect HA degradation in clinical practice and should be taken into consideration. Lastly, as mentioned, HYAL itself can help the spread of fluid in human tissues and exhibits its effect for longer than 1 hour.

## CONCLUSIONS

This study demonstrates that 300 U of HYAL is sufficient to degrade 0.2 mL of the most resistant HA fillers within 1 hour, offering a clearer guideline for clinicians treating HA-related complications. Additionally, a minimum dilution of 3:1 (liquid-to-filler ratio) should be used to provide adequate fluid for dissolving filler. Lastly, ovine HYAL appears to be just as effective as recombinant HYAL in terms of dissolving product.
